# Real-World Testing Practices, Treatment Patterns and Clinical Outcomes in Patients from Central Eastern Europe with EGFR-Mutated Advanced Non-Small Cell Lung Cancer: A Retrospective Chart Review Study (REFLECT)

**DOI:** 10.3390/curroncol29080460

**Published:** 2022-08-17

**Authors:** Urška Janžič, Nina Turnšek, Mircea Dediu, Ivan Shterev Donev, Roxana Lupu, Gabriela Teodorescu, Tudor E. Ciuleanu, Adam Pluzanski

**Affiliations:** 1 Medical Oncology Department, University Clinic of Respiratory and Allergic Diseases, 4204 Golnik, Slovenia; 2 Medical Oncology Department, Institute of Oncology Ljubljana, 1000 Ljubljana, Slovenia; 3 Sanador Oncology Center, 010991 Bucharest, Romania; 4 Department of Medical Oncology, MHAT Nadezhda, 1330 Sofia, Bulgaria; 5 Medical Department, AstraZeneca Romania, 013713 Bucharest, Romania; 6 Medical Oncology Department, Oncology Institute Prof. Dr. Ion Chiricuta, 400015 Cluj-Napoca, Romania; 7 Lung Cancer and Chest Tumours Department, Maria Sklodowska-Curie National Research Institute of Oncology, 02-781 Warsaw, Poland

**Keywords:** real-world retrospective study, advanced non-small cell lung cancer, EGFR *T790M* mutation

## Abstract

The targeted therapy with tyrosine kinase inhibitors (TKIs) against the epidermal growth factor receptor mutation (EGFRm) in advanced non-small cell lung cancer (NSCLC) changed the treatment paradigm. REFLECT study (NCT04031898) explored *EGFR*/*T790M* testing and treatment patterns in EGFRm NSCLC patients receiving first- or second-generation (1G/2G) EGFR TKIs as front-line (1L) in eight countries. Pooled data from Central Eastern Europe (CEE) countries from this study (Bulgaria, Poland, Romania, Slovenia) are presented here. This physician-led chart review study was conducted in patients with confirmed-EGFRm NSCLC initiating 1L 1G/2G EGFR TKIs between 2015–2018. The CEE cohort included 389 patients receiving 1L erlotinib (37%), afatinib (34%), and gefitinib (29%). Overall, 320 (82%) patients discontinued 1L, and 298 (77%) progression events were registered. Median progression free survival on 1L TKIs was 14.0 (95% CI: 12.6–15.6) months. Median overall survival from 1L start was 26.6 (95% CI: 24.1–29.0) months. Attrition rate between 1L and next line was 30%. Among patients with 1L progression, 200 (67%) were tested for *T790M* and 58% were positive. This first CEE analysis of treatments and outcomes in EGFRm NSCLC patients highlights the importance of using the most efficacious therapies currently available in 1L to reduce attrition and improve patient outcomes.

## 1. Introduction

Lung cancer is the most frequently diagnosed cancer worldwide, and around 85% of cases are represented by non–small cell lung cancer (NSCLC), a heterogeneous class of tumors [1]. Lung cancer-related mortality varies substantially worldwide. In 2015, the mortality rates in Eastern Europe were higher as compared to Western Europe and Scandinavia (45–63 per 100,000 versus 27–36 and 15–23 per 100,000, respectively) [2]. However, in 2020, a favorable reduction trend was observed in Central Eastern European (CEE) countries (22.7 per 100,00) [3].

Epidermal growth factor receptor mutation (EGFRm) occurs in 10–15% of NSCLC cases in Europe [4,5]. In the last decade, molecular testing at the time of diagnosis and addition of targeted therapies against activating mutations represented major steps toward clinically meaningful benefits for patients with EGFRm NSCLC. First-line (1L) therapy with the first-generation (1G; erlotinib and gefitinib) and second-generation (2G; afatinib, dacomitinib) EGFR tyrosine kinase inhibitors (EGFR TKIs) have proven effective against tumors harboring EGFR activating mutations [6,7]. However, 50–60% of patients receiving 1G/2G EGFR TKIs develop resistance due to a secondary mutation EGFR *T790M* that occurs after around one year of treatment [8,9,10].

Osimertinib is a third-generation EGFR TKI [11] that inhibits both *EGFR* activating and EGFR *T790M* resistance mutations. It has also demonstrated great potency in treatment of EGFRm NSCLC patients with central nervous system (CNS) metastases [12,13,14,15]. In 2016, osimertinib received the European Medicines Agency (EMA) approval for the treatment of patients with advanced/metastatic EGFR *T790M* mutation-positive NSCLC, based on AURA studies [16,17]. In 2018, it was approved as 1L treatment of advanced/metastatic EGFRm NSCLC based on the phase III FLAURA study and is currently the preferred front-line treatment [13,18,19,20].

Randomized clinical trial data suggest that 36% of patients who progressed on 1G/2G EGFR TKIs did not receive systemic second-line (2L) therapy [21], whereas real-world attrition rates from 1L to 2L in advanced/metastatic EGFRm NSCLC patients range from 10% to 62% [21,22,23,24]. These high attrition rates highlight the need for improved routine diagnostic practices and treatment strategies for better clinical outcomes, especially in countries with high lung cancer-related mortality. In CEE countries, drug accessibility and national reimbursement policies usually limit the access to the newest therapies. Therefore, treatment opportunities may differ from those in other European countries, which might impact the outcomes to some extent.

We conducted a retrospective chart review study in seven countries in Europe and Israel to describe treatment patterns of EGFRm NSCLC patients before the approval of osimertinib in 1L setting (clinicaltrials.gov: NCT04031898) [24]. Considering the limited information available on diagnostic practices, treatment patterns, and clinical outcomes in EGFRm NSCLC patients in CEE, here we describe the pooled data from four countries in the region participating in this retrospective study (Bulgaria, Poland, Romania, and Slovenia).

## 2. Materials and Methods

### 2.1. General Study Design and Data Source

The design of “The Real-world treatment patterns, clinical outcomes, and *EGFR*/*T790M* testing practices in EGFR-mutated advanced non–small cell lung Cancer patients receiving First-Line EGFR TKI Therapy” (REFLECT,) study was previously described in detail [24]. Briefly, REFLECT was a retrospective, non-interventional, multi-center, physician-led medical record review study conducted in Austria, Bulgaria, Greece, Poland, Romania, Slovenia, Switzerland, and Israel.

Of the 49 sites participating, 25 were from the CEE countries (results presented here). The medical oncologists and pulmonologists—who were responsible for treatment decisions for EGFRm NSCLC patients at each study center—reviewed the medical records of eligible consecutive patients receiving 1L 1G/2G EGFR TKIs between 1 January 2015, and 30 June 2018. Data were collected in a secured web-based data capture system.

Study approvals and waiver of informed consent forms were obtained from National and/or local Ethics Committees in all participating countries.

### 2.2. Study Objectives

Primary objectives were the proportion of 1G/2G EGFR TKIs used as 1L therapy in patients with EGFRm NSCLC, real-world progression free survival (rwPFS) on 1L and treatment strategies in patients receiving 2L therapy.

As secondary objectives, we described the demographic and baseline disease characteristics in patients with EGFRm NSCLC, *EGFR,* and *T790M* mutation testing practices, and treatment strategies in patients receiving third- (3L) or later-line therapies. We also assessed the proportion of patients with CNS metastases at the start and during treatment. Additionally, we evaluated attrition rates and overall survival (OS) from the start of 1L EGFR TKI therapy.

### 2.3. Study Population

Adult patients (age ≥ 18 years) with confirmed diagnosis of locally advanced/metastatic EGFRm NSCLC were identified in chronological order of starting the 1L EGFR TKI therapy, which was required to have been started between 1 January 2015, and 30 June 2018. Patients could have been alive or deceased at the time of medical record review.

Exclusion criteria included enrolment in a clinical trial assessing investigational therapies for EGFRm NSCLC, prior systemic therapies for advanced/metastatic disease other than EGFR TKI, and missing or unknown key study dates such as date of initial NSCLC diagnosis, date of first progression to advanced/metastatic disease, date of 1L EGFR TKI initiation, and date of death or last available follow-up.

The overall study index date was defined as the start date of the 1L 1G/2G EGFR TKI for advanced/metastatic EGFRm NSCLC. The data were collected from the initial diagnosis of NSCLC until death or last available medical record at the time of the medical chart review. Censoring was applied to the patients known to be alive at last available follow-up.

### 2.4. Variables and Epidemiological Measurements

The data collected were part of the general oncological assessments and management of the EGFRm NSCLC patients per routine clinical practice and national/local protocols and guidelines.

Real-world progression was defined as the radiological progression per any imaging method, start of new therapy line, death, or clinical progression as evaluated by the physician.

### 2.5. Study Outcomes

Primary outcomes: proportion of patients receiving either afatinib, gefitinib, erlotinib in 1L treatment; proportion of patients with disease progression on 1L EGFR TKIs; rwPFS on 1L EGFR TKIs; proportion of patients who received 2L therapy after progression on 1L therapy, and type of 2L therapy received.

Secondary outcomes: baseline patient demographics and disease characteristics; testing procedures for *EGFR* mutations; proportion of *T790M* testing; proportion of patients progressing on 2L and subsequent lines and type of treatments; proportion of patients with CNS metastases at metastatic disease diagnosis; proportion of patients who developed CNS metastases during treatment; OS from the start of 1L EGFR TKI.

### 2.6. Statistical Analysis

Study analyses were performed on datasets collected from all eligible patients. We descriptively analyzed the demographic and clinical characteristics, *EGFR* and *T790M* testing, and treatment patterns, by using frequencies and proportions for categorical variables and the mean, standard deviation, median, and range for continuous variables. A 95% confidence interval (CI) was derived for point estimations. We applied Kaplan-Meier methods to estimate the median rwPFS and median OS with 95% CI.

## 3. Results

### 3.1. Study Population

#### 3.1.1. Site Characteristics

Across CEE countries, 36% (9/25) of study sites were regional/national cancer centers, 32% (8/25) teaching/academic university hospitals, 20% (5/25) private hospitals or clinics, and 12% (3/25) general hospitals. In-house genetic testing services were provided by 44% centers, while 56% referred the NSCLC patients to different genetic testing laboratories. Reflex testing for *EGFR* at the time of the advanced/metastatic disease diagnosis was performed in half of the participating sites (*n* = 13; 52%), and reflex *T790M* mutation testing at the time of progression in one third of sites (*n* = 9; 36%). All the other centers performed on-demand testing.

#### 3.1.2. Disposition of Patients

Of the 390 EGFRm NSCLC patients enrolled into the CEE cohort, one was not eligible for inclusion in the analysis due to 1L EGFR TKI therapy start outside the study period, leaving 389 medical records reviewed across the CEE countries and distributed as follows: 31% (120/389) in Slovenia, 28% (110/389) in Poland, 23% (90/389) in Romania, and 18% (69/389) in Bulgaria.

#### 3.1.3. Baseline Patient Demographics and Disease Characteristics

The median age of patients included in the CEE cohort was 68 years, and 69% were female. Most patients had metastatic disease, adenocarcinoma, and ECOG performance status (PS) 0–1 at the time of initial NSCLC diagnosis. Details on patient demographics at baseline and disease characteristics are shown in Table 1. The median duration of follow-up between the initiation of 1L EGFR TKI therapy and last known date of follow-up or death was 21.4 months (min: 0.7; max: 58.9).

### 3.2. EGFRm Testing Patterns

The median time between initial diagnosis and EGFRm testing was 0.5 months across CEE centers. Initial EGFRm was tested from tissue biopsy (*n* = 324; 83%), cytology specimens (*n* = 57; 15%) or liquid biopsy (*n* = 8; 2%). EGFRm testing was performed from the primary tumor site in 76% of cases, secondary tumor site in 21% of cases, and unspecified for 3% of patients. Half of patients (52%) tested positive for the exon 19 deletion mutation, one third (34%) for exon 21 L858R point mutation, and the remaining patients (14%) for uncommon *EGFR* variants (Table 1).

### 3.3. NSCLC Treatment Patterns and Attrition Rates

#### 3.3.1. First-Line EGFR TKI Treatment Characteristics

**Figure 1:**
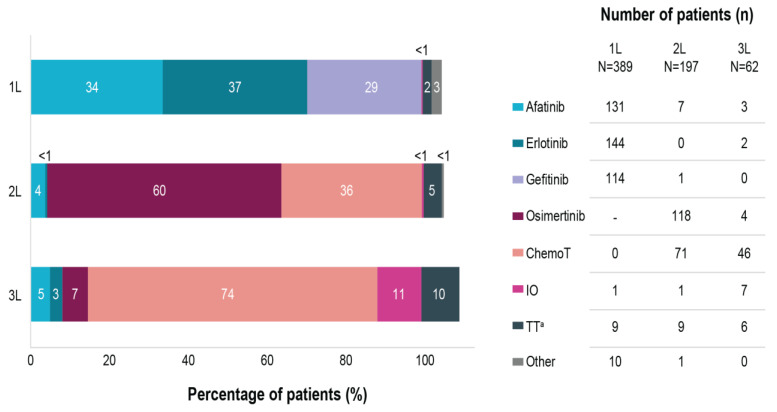
Treatment distribution in EGFRm NSCLC patients across first-, second- and third-lines of therapy. Note: multiple therapies could have been selected in one patient. ^a^ targeted therapy besides afatinib, erlotinib or gefitinib. 1L, first line therapy; 2L, second line therapy; 3L, third line therapy; N, number of patients initiating each therapy line; n, number of patients receiving specific treatments; ChemoT, chemotherapy; EGFRm, epidermal growth factor receptor mutation; IO, immunotherapy; NSCLC, non-small cell lung cancer; TT, targeted therapy; -, not applicable at the time of our study.

Erlotinib, afatinib, and gefitinib were used as 1L therapy in 37%, 34%, and 29%, respectively. In 5% of cases, other systemic treatments were administered in combination with the 1L EGFR TKI (Figure 1).

**Figure 2:**
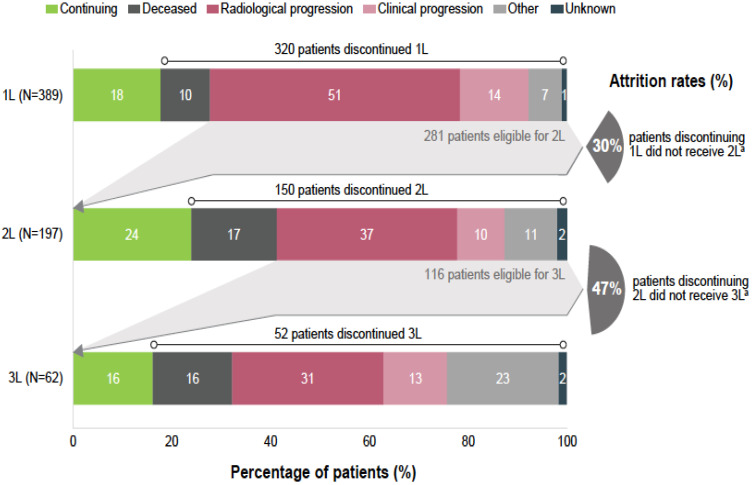
Reasons to discontinue first-, second- and third-line of therapy and attrition rates. Notes: Due to rounding, percentages may not always be 100%. ^a^ Deceased patients on each specific line have been excluded from the count. 1L, first line therapy; 2L, second line therapy, 3L, third line therapy; N, number of patients initiating each therapy line.

At the time of data collection, 82% (320/389) of patients discontinued the 1L EGFR TKI therapy, including 10% (39/389) of deaths on 1L EGFR TKI (Figure 2), whereas 18% (69/389) of patients continued 1L therapy. Discontinuation due to adverse events, which were included in the category other reasons, occurred in 5% (20/389) of cases. In total, 77% (298/389) per-protocol progression events on 1L were reported, which included radiological progression (*n* = 197; 51%), clinical progression (*n* = 54; 10%), death (*n* = 39; 10%) and start of a new line therapy without having a documented disease progression (*n* = 8; 2%).

Median rwPFS on 1L EGFR TKI across the pooled CEE cohort was 14.0 (95% CI: 12.6; 15.6) months, with the following results at country level: Bulgaria 16.3 (95% CI: 11.5; 22.6) months, Poland 12.9 (95% CI: 10.3; 15.4) months, Romania 12.0 (95% CI: 10.3; 15.6) months, and Slovenia 15.6 (95% CI: 12.6; 19.2) months, respectively.

At the end of the data collection period, 225 (58%) deaths had been recorded. Median OS from the start of 1L EGFR TKI therapy was 26.6 (95% CI: 24.1; 29.0) months across the entire CEE cohort, with the following results by country: Bulgaria 23.4 (95% CI: 18.2–20.2) months, Poland 26.2 (95% CI: 18.0; 29.7) months, Romania 26.4 (95% CI 22.4; 34.2) months, and Slovenia 28.9 (95% CI: 25.0; 34.3) months, respectively.

#### 3.3.2. Second- and Subsequent Treatment Patterns and Attrition Rates

Among patients discontinuing 1L EGFR TKI, 62% (197/320) received 2L therapy. Excluding patients deceased during 1L treatment, 30% (84/281) of the patients discontinuing the EGFR TKI therapy did not receive any further treatment (Figure 2). Osimertinib was the most common treatment choice in 2L, being reported for 60% (118/197) of patients initiating treatment after progression on 1L (Figure 1), equivalent of 40% (118/298) of all patients with a per-protocol progression event on 1L. It should be noted that all participating sites had access to 2L treatment with osimertinib either through formal reimbursement or via patient named programs. At the time of data collection, 2L therapy was continued only by 24% (47/197) of patients, whereas the rest discontinued 2L, mostly due to radiological progression (*n* = 72; 37%). Excluding patients deceased during 2L, 47% (54/116) of patients discontinuing 2L therapy did not receive any further treatment (Figure 2).

Among patients discontinuing 2L, 41% (62/150) received 3L therapy. Chemotherapy was the most common treatment choice, being administered in 74% (46/62) of patients initiating 3L (Figure 1). At the time of data collection, 3L therapy was continued for only 16% (10/62), whereas 84% (52/62) of patients discontinued 3L, mostly due to radiological progression (*n* = 19; 31%) (Figure 2). Excluding patients deceased on 3L, 67% (28/42) of patients discontinuing 3L therapy did not receive any further treatment.

Among patients discontinuing 3L, 27% (14/52) received 4L therapy, mainly with chemotherapy (*n* = 8), osimertinib (*n* = 4) and targeted therapy (gefitinib, *n* = 4). At the time of data collection, 4L therapy continued only for two patients; the rest discontinued the treatment due to radiological or clinical progression (*n* = 8; 57%), death (*n* = 3), and unknown reason (*n* = 1). At the time of data collection, only five patients received 5L therapy, consisting mainly in targeted therapy (erlotinib or capmatinib; *n* = 3), chemotherapy (*n* = 1) and osimertinib (*n* = 1). All patients discontinued 5L therapy at the time of data review due to radiological or clinical progression (*n* = 3), death (*n* = 1), and deteriorating condition (*n* = 1).

### 3.4. T790M Mutation Testing and Treatment Patterns

The median time between initiation of 1L EGFR TKI and testing for *T790M* mutation was 12.8 months (min: −1.7; max: 51.2). Cobas^®^ *EGFR* mutation test was used in two-thirds of cases (*n* = 134; 67%), while for the rest of cases, the test was reported as other or unknown.

In patients progressing on 1L EGFR TKI, 67% (200/298) were tested for the presence of *T790M* mutation at any time, with positive results reported in 58% (115/200) of patients tested. *T790M* mutation testing was performed using liquid biopsy (*n* = 150; 75%), tissue biopsy (*n* = 34; 17%), cytology (*n* = 15; 8%) and unknown specimen for one patient. In patients progressing on 1L EGFR TKI and positive *T790M* mutation, 97% (111/115) received osimertinib in any subsequent line of therapy, but mostly in 2L (*n* = 107;93%). Osimertinib was also administered to 15% (9/85) of patients with 1L TKI progression and negative *T790M* test results (most in 2L and for 1 patient in 3L), and to 3% (3/98) of progressing patients without any *T790M* testing recorded.

### 3.5. Patients with CNS Metastases

One third (*n* = 127; 33%) of the patients from the CEE cohort had CNS metastases recorded in medical chart either at the start of 1L EGFR TKI therapy (*n* = 83; 21%) or at any time after starting the front-line therapy (*n*= 44; 11%). The median age of NSCLC patients at first diagnosis of CNS metastases was 66.0 years (min: 33.0; max: 89.0), most were female (*n* = 84; 66%).

For patients with CNS metastases developed during treatment, the median time between the start of 1L EGFR TKI therapy and first diagnosis of the brain lesion was 18.5 months (min: 1.9; max: 53.8 months). CNS metastases diagnosis was confirmed by either imaging methods (MRI, CT scan, etc.) (*n* = 122; 96%), tissue biopsy (*n* = 12; 9%), or both. Treatments of CNS metastases included whole brain radiation therapy (58%), targeted therapy (27%), stereotactic radiosurgery (21%), surgical resection (12%), chemotherapy (2%), and other unspecified treatment (2%), whereas 8% of patients did not receive any treatment for the CNS lesions.

In patients with CNS metastases at the start of 1L EGFR TKI therapy, the median OS from the diagnosis of the first CNS metastasis was 21.8 (95% CI: 18.1; 24.9) months, while in those who developed CNS metastases during the treatment, the median OS was 6.4 (95% CI: 3.5; 10.2) months.

## 4. Discussion

We provide a comprehensive overview of the real-world management of patients with advanced/metastatic EGFRm NSCLC and clinical outcomes following 1L 1G/2G EGFR TKIs therapies in four countries from the CEE region. Our analysis was performed on data from the REFLECT study, a large multi-national retrospective chart review conducted in eight countries (*n* = 896) [24]. In the CEE cohort, three out of four patients with EGFRm NSCLC experienced disease progression (74%) on 1L 1G/2G EGFR TKI therapy after a median of 14 (95% CI: 12.6; 15.6) months. In patients with progression on 1L EGFR TKI, one third of patients was not tested for *T790M* resistance mutation (33%). Across the entire cohort, one third of patients discontinuing 1L therapy did also not receive any further treatment (30%), and the attrition rates increased in later lines.

Demographic and disease characteristics of patients from this CEE cohort were similar to those reported by other real-world studies conducted in European metastatic EGFRm NSCLC populations [25,26,27,28,29]. In our cohort, the median age at inclusion was 68 years, most of the cases were recorded in female patients and never-smokers, with an ECOG PS of 0–1, metastatic disease, and adenocarcinoma histology at initial diagnosis. The 1G/2G EGFR TKIs—erlotinib, afatinib, and gefitinib—were administered as 1L treatment per ESMO recommendations at the time of the study [30]. Disease progression on 1L TKI was recorded in 77% of patients from the CEE cohort versus 81% in the full analysis set [24]. Similar progression rates have been recorded in other real-world evidence studies with 1L 1G/2G EGFR TKIs, i.e., 74% in Belgium and 73% in a United States (US)/European study, and slightly higher in national studies conducted in Germany (84%) and the US (85%) [22,23,25,26].

Our cohort analysis revealed that only two-thirds of patients progressing on 1L 1G/2G EGFR TKI (67%) were tested for the presence of *T790M* mutation, slightly less than in the overall study cohort (71%) [24]. Other studies conducted during the same period reported testing rates of 72% to 86% [23,25,26]. In both CEE and overall REFLECT cohorts, the *T790M* positivity rates among patients progressing on 1L was 58% [24], while in other studies the positivity rates ranged from 48% to 71% [23,25,26,27,28]. Since 50–60% of tumor progressions on 1L EGFR TKI are caused by the occurrence of the *T790M* resistance mutation [8,9,10], the number of patients with a potential benefit from osimertinib as 2L therapy when *T790M* mutation is present would have been higher if all progressing patients from our cohort would have been tested. Unsatisfactory testing rate reflects routine practice in CEE countries. Reflex *T790M* testing for all EGFRm NSCLC patients progressing on 1G/2G EGFR TKI is of utmost importance for further treatment strategy, because no well-defined clinical characteristics are representative for the development of this resistance mutation [31,32].

The attrition rates between 1L and 2L and 2L and 3L (excluding patients deceased during treatment) were 30% and 47%, respectively. Likewise, several real-world studies reported attrition rates following 1L treatment of EGFRm NSCLC of 22–37% and 43% after 2L therapy [23,26,27]. As suggested in the published literature, these high attrition rates are most likely the result of accelerated deterioration of patient’s clinical condition, lack of *T790M* mutation testing availability including of liquid biopsy, and no patient’s agreement for another invasive diagnostic procedures or repeated biopsies [21,23]. The low percentage of *T790M* mutation testing recorded in CEE countries was probably due to the lack of reimbursement of liquid biopsies in Poland, Bulgaria, and Romania at the time of our study [33], impacting the availability of patients for 2L treatment with osimertinib. In addition, the *T790M* reflex testing at progression was performed in one third (36%) of sites, a suboptimal finding that probably has changed over time as technology improved, and liquid biopsies at progression are adequate methods, despite low sensitivity [8]. Presumably, the attrition rates in CEE countries decreased over time following improved *T790M* testing and reimbursement of third-generation osimertinib [8,20].

While our analysis is the first across the CEE region documenting the clinical outcomes and attrition rates on 1L 1G/2G EGFR TKIs, it brings evidence applicable only to the pre-FLAURA era. The preliminary reports of the first real-world studies with front-line osimertinib in advanced/metastatic EGFRm NSCLC are in line with the improved survival data reported in randomized clinical studies [34,35]. The median rwPFS on 1L 1G/2G EGFR TKIs recorded across the CEE cohort was of 14.0 months, being slightly higher than in the primary REFLECT study, where it was 12.6 months [24]. However, rwPFS at country level in this CEE cohort varied, ranging from 12.0 months in Romania and 12.9 months in Poland to 15.6 months in Slovenia and 16.3 months in Bulgaria, differences probably explained by patients’ heterogeneity and local characteristics of the medical care. In other real-world studies that included patients with similar demographic characteristics, the median rwPFS was 11.9 months in Poland, and only 7.6 months in Belgium [26,29]. Results recorded in Belgium were probably influenced by a higher percentage of patients with ECOG PS 3–4 (16% versus 3% in our study, respectively), which led to a higher loss of patients after the 1L therapy [26]. The median OS from the start of 1L EGFR TKI therapy was 26.6 months in our cohort, similar to the OS reported in the primary REFLECT study (26.2 months) [24]. Other real-world studies on similar EGFRm NSCLC populations treated with 1L 1G/2G EGFR TKI reported results ranging from 19.4 to 27.4 months [24,25,26,28]. The variability might be due to a different distribution of 1G/2G EGFR TKIs used in the 1L treatment across these countries, and other clinical and local healthcare-related factors.

Similar to the primary cohort study, and other data published in the literature, one third of patients had CNS metastases either at the start of EGFR TKI treatment as 1L or during the treatment, with lower OS as compared to patients without CNS involvement [36,37,38]. These findings support the need of using efficacious therapies for CNS metastases as early as possible in the EGFRm NSCLC treatment pathway. Current ESMO guidelines recommend the use of CNS-penetrant next generation EGFR TKIs to control the metastases and to delay cranial radiotherapy in these patients [8,20]. Osimertinib has demonstrated systemic efficacy in patients with untreated EGFRm NSCLC, being able to penetrate the blood brain barrier and to achieve a significant exposure in the CNS compared to other EGFR TKIs [15,39].

Our study strength is in its real-life character. The minimum set of inclusion and exclusion criteria allowed us to build real-world evidence coming from the four CEE countries. Yet, the number of patients was restricted considerably because no prior systemic therapy for advanced/metastatic disease was allowed, other than EGFR TKIs, and in some countries, chemotherapy was often used due to delays in EGFR TKIs authorizations by the Health Insurance Houses during 2015–2017. Due to these small numbers of patients, the generalizability of the results across countries or at country level is further hindered. In addition, the observational and retrospective design is restrictive, because it implies missing data or coding errors in the available medical charts. However, to meet the primary objectives, several data were mandatory for data collection. Another limitation is the descriptive nature of the analysis, without additional stratifications by treatments at baseline, stages, and histology types. This study had no formal hypothesis on the effectiveness of EGFR TKIs and was not powered for comparisons between individual therapies and countries, this being the main reason why it only provides an overall overview on the diagnosis practices, treatment patterns, and clinical outcomes in CEE countries.

## 5. Conclusions

This is the first analysis of a cohort of patients with EGFRm NSCLC receiving 1G/2G EGFR TKIs in 1L therapy from four CEE countries, and it offers a comprehensive overview of treatment patterns and clinical outcomes. We found that only one of five patients was continuing 1L 1G/2G EGFR TKIs treatment at the time of data collection, while one of three patients discontinuing 1L did not receive any further treatment. Given these high attrition rates, our analysis highlights the importance of reflex testing and timely identification of patients with actionable mutations from initial diagnosis of advanced/metastatic disease. This would enable the use of most efficacious treatments currently available in front-line setting with proven positive impact on patient outcomes.

## Figures and Tables

**Table 1:** Patient demographics at baseline and disease characteristics at initial advanced/metastatic NSCLC diagnosis (CEE cohort).

Category	NSCLC Patients *n* = 389
Age (years); median (min; max) ^a^	68.0 (33.0; 93.0)
Sex (female); *n* (%)	268 (69)
Smoking status; *n* (%)	
Never smoker	200 (51)
Former smoker	89 (23)
Current smoker	26 (7)
Unknown	74 (19)
Initial disease stage, *n* (%)	
Early stage (I–II)	33 (9)
Limited regional (IIIA)	13 (3)
Locally advanced (IIIB)	19 (5)
Metastatic (IV)	323 (83)
Not known	1 (0)
Tumor histology, *n* (%)	
Adenocarcinoma	367 (94)
Squamous cell carcinoma	8 (2)
Mixed histology	7 (2)
Other	7 (2)
ECOG performance status, *n* (%)	
0	83 (21)
1	234 (60)
2	45 (12)
3	8 (2)
4	1 (<1)
Not known	18 (5)
Site of distant metastases cases, *n* (%) ^b^	
Lung	196 (50)
Bone	134 (34)
Lymph nodes	127 (33)
Pleura	127 (33)
Brain	79 (20)
Liver	57 (15)
Adrenal	39 (10)
Other ^c^	32 (8)
*EGFR* mutations, *n* (%)	
*ex19del*	202 (52)
*L858R*	134 (34)
Other	53 (14)
*G719X*	12 (3)
*G719X* + *L861Q*	2 (1)
*G719X* + *S768I*	4 (1)
*L858R* + *S768I*	1 (<1)
*L858R* + *T790M*	2 (1)
*L861Q*	10 (3)
*S768I*	1 (<1)
*ex20ins*	5 (1)
Other/not specified	16 (4)

^a^ first diagnosis of locally advanced/metastatic NSCLC; ^b^ in patients with locally advanced unresectable or metastatic NSCLC diagnosis; ^c^ other included bone marrow (<1%), eye (1%), kidney (1%), pancreas (<1%), pericardium (2%), peritoneum (1%), skin/soft tissue (1%), spleen (1%) and pleural effusion (2%); NSCLC, non-small cell lung cancer; *n*, number of patients included in the analysis; min, minimum; max, maximum; n, number of patients in a given category; ECOG, Eastern Cooperative Oncology Group; EGFR, epidermal growth factor receptor; ex19del, exon 19 deletion; ex20ins, exon 20 insertion.

## Data Availability

The data presented in this study are available on request from the study sponsor.
